# Increased serum Interleukin-18 in the active phase of Henoch-Schönlein purpura

**DOI:** 10.1186/1546-0096-9-S1-P83

**Published:** 2011-09-14

**Authors:** Donato Rigante, Anna Zampetti, Giulia Bersani, Beatrice Bartolomei, Claudio Feliciani, Achille Stabile

**Affiliations:** 1 Department of Pediatric Sciences, Università Cattolica Sacro Cuore, Rome, Italy; 2 Department of Dermatology, Università Cattolica Sacro Cuore, Rome, Italy

## Introduction

Henoch-Schönlein purpura (HSp) is the most frequent systemic vasculitis occurring in childhood with typical skin involvement and concurrent signs involving joints, gastrointestinal tube and kidney. HSp pathogenesis is still undefined though a multifacet cytokine web is believed to contribute in its biologic process.

## Aim

To establish the relationship between serum levels of interleukin (IL)-18 and disease outcome and assess its feasibility to provide a marker of disease activity in the clinical practice.

## Patients/methods

We evaluated clinical/laboratory variables and serum IL-18 in 17 unselected children (13 males, 4 females; mean age at the onset: 7.5±3.4 years; age range: 3-16 years) hospitalized during the last year for HSp, diagnosed by EULAR/PRINTO/PRES criteria; the same patients were re-evaluated after 6 months too. All results were compared with 25 age-matched healthy controls. IL-12 and IL-6 were also evaluated in a cohort of the same patients and compared with controls.

## Results

Groups were compared through non parametric tests: Wilcoxon signed rank test for unpaired data or Mann-Whitney U test for paired data. Logistic regression was used to compare serum IL levels with general/clinical variables (sex, edema of hands/feet, gastrointestinal or renal complications, relapses and renal involvement at 6 months). Serum IL-18 levels showed no relationship with general/clinical variables, but appeared significantly higher at disease onset in comparison with healthy controls and significantly reduced after 6 months: respectively 579.1 ± 488.1 (range: 162.7- 2224.45) and 369.8 ± 193.8 (range: 118.15-744.9). Conversely, IL-12 levels at onset were similar to controls, but significantly higher after 6 months, whilst IL-6 levels were significantly higher both at onset and after 6 months than in controls.

## Conclusions

The significant increase of serum IL-18 in the acute phase of HSp might suggest its direct involvement in the induction of the inflammatory process. Though preliminary and expecting further confirmation on a larger sample these data support the conclusion that serum IL-18 levels reflect HSp activity.

